# *Edwardsiella ictaluri* T3SS Effector EseN Modulates Expression of Host Genes Involved in the Immune Response

**DOI:** 10.3390/microorganisms10071334

**Published:** 2022-07-01

**Authors:** Lidiya P. Dubytska, Ranjan Koirala, Azhia Sanchez, Ronald Thune

**Affiliations:** 1 Department of Biology and Chemistry, Southern University and A & M College, Baton Rouge, LA 70813, USA; koirala.ranjan@sus.edu (R.K.); azhia.sanchez@sus.edu (A.S.); 2 Department of Pathobiological Sciences, Louisiana State University School of Veterinary Medicine, Baton Rouge, LA 70803, USA; rthune@lsu.edu; 3 School of Animal Science, Louisiana State University Agricultural Experiment Station, Baton Rouge, LA 70803, USA

**Keywords:** *Edwardsiella ictaluri*, type III secretion system effectors, EseN, ERK1/2, cytokines, transcription factors, prostaglandin, channel catfish, *Ictalurus punctatus*

## Abstract

The type III secretion system (T3SS) effector EseN is encoded on the *Edwardsiella ictaluri* chromosome and is homologous to a family of T3SS effector proteins with phosphothreonine lyase activity. Previously we demonstrated that *E. ictaluri* invasion activates extracellular signal-regulated kinases 1 and 2 (ERK1/2) early in the infection, which are subsequently inactivated by EseN. Comparative transcriptomic analysis showed a total of 753 significant differentially expressed genes in head-kidney-derived macrophages (HKDM) infected with an EseN mutant (∆EseN) compared to HKDM infected with wild-type (WT) strains. This data strongly indicates classical activation of macrophages (the M1 phenotype) in response to *E. ictaluri* infection and a significant role for EseN in the manipulation of this process. Our data also indicates that *E. ictaluri* EseN is involved in the modulation of pathways involved in the immune response to infection and expression of several transcription factors, including NF-κβ (c-*rel* and *relB*), *creb3L4*, *socs6* and *foxo3a*. Regulation of transcription factors leads to regulation of proinflammatory interleukins (IL-8, IL-12a, IL-15, IL-6) and cyclooxygenase-2 (COX-2) expression. Inhibition of COX-2 mRNA by WT *E. ictaluri* leads to decreased production of prostaglandin E2 (PGE2), which is the product of COX-2 activity. Collectively, our results indicate that *E. ictaluri* EseN is an important player in the modulation of host immune responses to *E.ictaluri* infection.

## 1. Introduction

*Edwardsiella ictaluri* (*E. ictaluri*) is a Gram-negative bacterium of the family Enterobacteriaceae that infects and causes enteric septicemia (ESC) of channel catfish (*Ictalurus punctatus*). Central to *E. ictaluri* pathogenesis is a type III secretion system (T3SS) [1]. A recent report provided the identification and characterization of nine T3SS effector proteins of the *E. ictaluri* T3SS that are translocated from bacteria in the *Edwardsiella*-containing vacuole (ECV) through the vacuolar membrane to the host cell cytoplasm [2]. It was reported that in many pathogens secreted effectors can affect intracellular uptake, surface colonization of the cell without uptake, adherence to macrophages and inhibition of phagocytosis, cytotoxicity, vesicular trafficking, control of programmed cell death, manipulation of gene expression, and up- and down-regulation of inflammatory cytokines [3,4,5,6,7,8,9,10,11]. The *E. ictaluri* effector EseN is a phosphothreonine lyase (PTL) that inactivates extracellular mitogen-activated protein kinases (ERK1/2) [12]. Based on similarity to other effectors, EseN can also inactivate other mitogen-activated-protein-kinases (MAPKs) [13,14]. MAPK pathways are central signaling cascades that are essential for the activation of host innate immune responses [15]. Moreover, MAPKs can cooperate with other signal transduction pathways, including that of the transcriptional regulator nuclear factor-κβ (NF-κβ), which regulates the transcription of genes important for cytokine and chemokine production, including interleukin IL-6, IL-8, IL-12, and tumor necrosis factor-α [16,17,18], as well as the anti-apoptotic factor Bcl-2 [17,19]. Phosphorylation of MAPKs leads to activation or inhibition of transcription factors [15,20], resulting in up- or down-regulation of important immune factor genes that regulate bacterial infections in the host [13,21,22,23,24,25]. 

Several cytokines and eicosanoids, such as prostaglandins (PGs) and leukotrienes, are known to affect the function of macrophages [26]. The rate-limiting step in PG synthesis is catalyzed by cyclooxygenase (COX), which converts arachidonic acid to PGH2, the common precursor to all PGs [27]. There are two isoforms of COX enzyme that are encoded by distinct genes. The COX-1 is constitutively expressed in most cell types and plays a role in gastrointestinal and reproductive function, COX-2 is normally expressed at very low levels but is strongly induced by various stimuli, including mitogens, cytokines, hormones, and oncogenes [28].

Because EseN is important for the virulence of *E. ictaluri* [2,12] and the virulence mechanisms of this factor are unknown, RNAseq and transcriptome analysis were used to identify potential genes and pathways that are modulated by EseN. We used RT-qPCR to confirm RNAseq data and to study extended time points of infection. In this study, we also investigated the involvement of EseN in the modulation of COX-2 mRNA expression and PGE2 synthesis in macrophages infected with *E. ictaluri* strains. For the first time, we demonstrate that *E. ictaluri* T3SS effector EseN is involved in the M1-HKDM phenotype in response to infection. Our data also indicates that *E. ictaluri* EseN modulates pathways involved in immune responses to infection, expression of NF-κβ (*c-rel* and *relB*), *creb3L4, socs6*, and *foxo3a* transcription factors, that lead to the regulation of pro-inflammatory interleukins production (IL-8, IL-12a, IL-15, IL-6), COX-2 expression, and PGE2 synthesis. 

## 2. Materials and Methods

### 2.1. Bacterial Strains

*E. ictaluri* WT strain 93–146 and a ∆EseN mutant [12] were grown for 16–18 h at 28 °C to an OD_600_ = 1.8 to 2.0 in porcine-brain-heart-infusion (BHI) broth. All strains grown in broth were aerated on a Max Q4450 incubated shaker (Thermo Scientific, Marietta, OH, USA).

### 2.2. Infection Procedure

Isolation of HKDM was performed as previously described [29]. HKDM were seeded in 6 well plates for RNA preparation and in 24 well plates for detection of PGE2 production. For *E. ictaluri* infection, bacteria were opsonized for 30 min in normal autologous serum and added to duplicate (for RNA purification) or triplicate (for PGE2 detection) wells with HKDM cultures at a multiplicity of infection (MOI) of 10 bacterium to 1 HKDM. Uninfected cells were used as a negative control. After infection, plates were centrifuged at 500× *g* for 5 min to synchronize contact of the bacteria with the adhered cell layer and allowed to incubate for 30 min, after which 100 µg/mL gentamicin was added for 1 h at 28 °C to kill any remaining extracellular bacteria. Finally, wells were washed once with ctRPMI [29], after which ctCCMM [29] containing a 1 µg/mL bacteriostatic dose of gentamicin was used to control the extracellular growth of any bacteria released from the cells during the experiment. At 1 h, 3 h, 5 h, or 7 h, samples were collected for RNA prep or PGE2 detection experiments.

### 2.3. RNA Extractions and Sequencing

For RNA sequencing (RNAseq), total mRNA extraction was carried out on HKDM samples using the Dynabeads mRNA direct purification kit (Ambion, Austin, TX, USA) and analyzed for quality and purity by fragment analysis (Advanced Technologies, Inc., Newport News, VA, USA). The mRNA samples with ribosomal RNA contamination of less than 10% were used for the assay. The RNAseq runs were carried out by the LSU School of Veterinary Medicine Genelab Core. The mRNAs were sequenced using an Ion Proton NextGeneration Sequencer. For RT-qPCR total RNA extractions were carried out on HKDM samples using RNAzol^®^ RT Isolation Reagent (Molecular Research Center, Cincinnati, OH, USA) in combination with the Pure Link RNA mini-kit (Invitrogen, Carlsbad, CA, USA) that was used for DNAse treatment and washing steps only, following manufacturer protocols. Samples were resuspended in molecular-grade water (Ambion, TX, USA) and stored at −80 °C until use. The RNA concentration and purity were determined using Nanodrop (BioTek Synergy LX Multi-Mode Reader, Daytona Beach, FL, USA) with software Gen5 version 3.11.

### 2.4. RT-qPCR

The cDNA was prepared from 200 ng of total RNA using the SuperScript™ III First-Strand Synthesis System for RT-PCR (Invitrogen, Carlsbad, CA, USA) according to the procedures provided by the manufacturer. Quantitative PCR (qPCR) was performed in a 96-well plate with PowerUp™ SYBR™ Green Master Mix (Applied Biosystem by Life Technologies, CA, USA) and 0.5 μM of each gene-specific primer (Table 1) according to the manufacturer’s instruction in a LightCycler^®^ 96 System (Roche Applied Science, Indianapolis, IN, USA). Oligonucleotide primers were purchased from Integrated DNA Technologies (Coralville, IA, USA). The CanX and SDHA were used as reference genes. The following standard thermal profile was used for all qPCR reactions: uracil-DNA glycosylase (UDG) activation (50 °C for 2 min), polymerase activation (95 °C for 2 min), 40 cycles of amplification and quantification (95 °C for 15 s, 60 °C for 1 min), and dissociation curve (95 °C 15 s, 60 °C 1 min, 95 °C 30 s). In addition, qPCRs were carried out by qPCRBIO SyGreen 1-Step Go Lo-R kit (PCRBiosystems, Wayne, PA, USA). One-step qPCR was performed using 5 ng of respective RNA in each reaction mixture under conditions of 54 °C for 10 min, 95 °C for 2 min, and 50 cycles of 95 °C for 5 s and 61 °C for 30 s, followed by determination of the melting curve. Similar data were obtained in both cases.

### 2.5. The mRNAseq Data Analysis

Data was compiled and analyzed by the LSU Center for Computational Technology using Partek Flow based on researcher input. Unaligned reads were converted to FASTQ format, trimmed using a quality score and read length (Phred > 20; >25 bp), then aligned 55 to the *Ictalurus punctatus* reference genome using STAR (Version 2.6.1d) [30]. The reads were then quantified to the *Ictalurus punctatus* genome, calculating the fragments per kilobase of transcript per million aligned reads (FPKM) using the Partek Flow quantification algorithm, based on the hierarchical model described by Leng et al. [31]. Differential analysis between each macrophage treatment (WT versus uninfected, ΔEseN versus uninfected, and WT versus ΔEseN) was carried out using Gene Specific Analysis (GSA), and transcripts were selected by q and *p*-value of 0.05 or less.

The proteomic results were analyzed by multiple approaches. Database for Annotation, Visualization and Integrated Discovery (DAVID software; http://david-d.ncifcrf.gov, accessed on 20 June 2022) was used to classify the functional categories and gene ontology (GO) annotation enrichment analysis of differentially expressed genes (DEGs). The GO analysis was performed at three levels: Molecular function, biological process, and cellular component.

Pathway analysis was performed using the Kyoto Encyclopedia of Genes and Genomes database (KEGG) (www.genome.jp/kegg, accessed on 20 June 2022). Functional enrichment and pathway enrichment analyses were performed by using DAVID. *p*-value was adjusted by the method of Benjamini-Hochberg to control the false discovery rate. Enriched GO terms and KEGG pathways were identified as significant with *p* < 0.05.

### 2.6. Prostaglandin Assay

To measure PGE2 production, HKDM were seeded in 24-well cell culture plates in triplicate, cultured in ctCCMM, and infected with *E. ictaluri* WT and ∆EseN mutant strains as described above. Following post-infection at 1 h, 3 h, 5 h, and 7 h, the levels of PGE2 in the supernatants and lysates were determined using a PEG2 high-sensitivity ELISA kit (Enzo Life Sciences Inc, Farmingdale, NY, USA) according to manufacturer’s protocol. The results were expressed in pg/mL.

### 2.7. Statistical Analysis

The RT-qPCR assays were performed in triplicate, and relative expression was calculated by the normalized ratio obtained using LightCycler^®^ 96 Application Software. To improve this normalization step, CanX and SDHA were chosen as reference genes. In this case, each relative ratio is calculated separately, and the geometric average is displayed. Results are recalculated based on the relative ratio measured for each sample at the study calibrator condition, that is, the start of the experiment. Changes in gene expression and PGE2 production were analyzed using One-way Analysis of Variance (ANOVA) followed by Bonferroni procedure for comparison of group means. Comparison between two groups was also analyzed by *t*-test.

## 3. Results

### 3.1. Edwardsiella ictaluri T3SS Effector EseN Modulates Host Gene Expression

**Figure 1:**
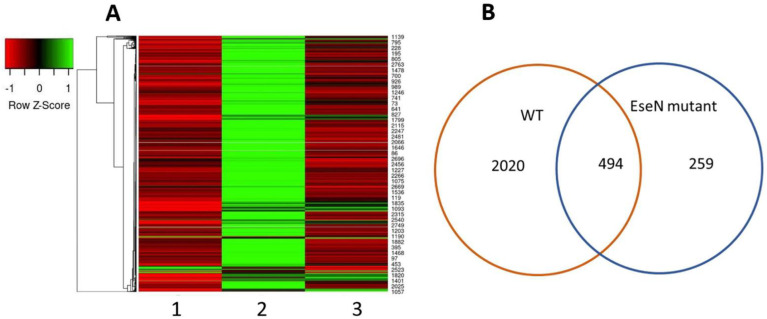
Differentially expressed genes from three independent RNAseq experiments using HKDM infected with WT *E. ictaluri* (HKDM-WT) as well as an *E. ictaluri* ΔEseN mutant (HKDM-ΔEseN). (**A**) Cluster analysis of 753 significant differentially expressed genes (by *p* and q values less than 0.05) in HKDM-∆EseN compared to HKDM-WT. 1. Uninfected HKDM, 2. HKDM-WT, 3. HKDM-∆EseN. (**B**) Comparative transcriptomic analysis of genes that were significantly differentially expressed in HKDM-WT compared to uninfected HKDM (WT), and HKDM-∆EseN compared to HKDM-WT (EseN mutant). A total of 494 of these genes were common for HKDM-∆EseN and HKDM-WT.

Comparative transcriptomic analysis showed a total of 753 significant differentially expressed genes (DEG) with *p* and q-values of 0.05 or less (Figure 1). Only 7 of 753 genes were upregulated in HKDM infected with ∆EseN (HKDM-∆EseN) compared to HKDM infected with WT (HKDM-WT). These genes include *ccnd3*, *arf4*, *h2B*-like, leucine-rich repeat-containing protein 15-like, *ehd1b*, *vamp5*, and *IL-12a*. Three of them can play an important role in vesicle-mediated transport (*arf4*, *ehd1b*, *vamp5*), and two of them in immune responses *IL-12a* and leucine-rich repeat-containing protein 15-like. Interestingly, in HKDM-WT expression of *ccd3*, *h2B*-like, *ehd1b*, and *IL-12* was not changed compared to uninfected HKDM, and expression of *arf4* and leucine-rich repeat-containing protein 15-like was increased. The rest of the 746 genes were downregulated in HKDM-∆EseN compared to HKDM-WT.

Interestingly, 492 of 753 genes were upregulated in HKDM-WT versus HKDM and were down regulated in HKDM-∆EseN mutant compared to HKDM-WT (Figure 1). A total of 254 genes were downregulated only in HKDM-∆EseN mutant strain compared to HKDM-WT, indicating that other *E. ictaluri* effectors or proteins complement this effect. In that same time, 2020 genes were up or down regulated only in HKDM-WT *E. ictaluri* compared to uninfected HKDM (Figure 1).

The 214 differentially expressed genes in HKDM-∆EseN mutant compared to HKDM-WT were initially selected as not significant because of the q value (q = 0.05–0.06). All of these genes were highly significant by *p* value (less than 0.05) therefore we selected some of these genes that contain interleukins and transcription factors for this study. The significance of these genes was confirmed by qPCR.

**Figure 2:**
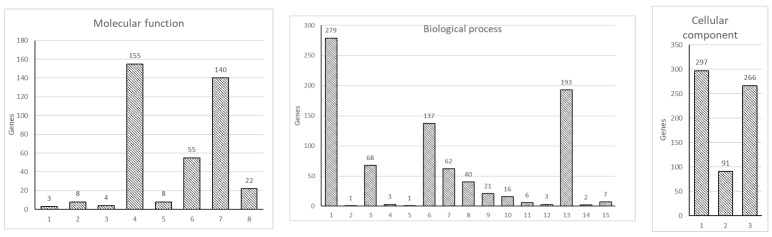
Gene ontology enrichment analysis of DEG. 753 DEG were analyzed for enrichment in three GO ontologies: biological process, cellular component, and molecular function. The number of genes enriched in individual GO terms is indicated on top of the individual bars. Molecular function: (1) translation regulator activity; (2) molecular transducer activity; (3) molecular adaptor activity; (4) binding; (5) structural molecule activity; (6) molecular function regulator; (7) catalytic activity; (8) transporter activity. Biological process: (1) cellular process; (2) reproductive process; (3) localization; (4) interspecies interaction between organisms; (5) reproduction; (6) biological regulation; (7) response to stimulus; (8) signaling; (9) developmental process; (10) multicellular organismal process; (11) locomotion; (12) biological adhesion; (13) metabolic process; (14) growth; (15) immune system process. Cellular component: (1) cellular anatomical entity; (2) protein-containing complex; (3) intracellular.

To further analyze the data, we carried out a GO enrichment analysis, which showed an enrichment in the three GO ontologies (molecular function, cellular component, and biological process) and multiple terms within individual GO ontology in DEGs between HKDM-∆EseN compared to HKDM-WT (Figure 2). Evidence suggests that these three GO ontologies are affected. Importantly, organelle development and movement, biological responses to stimulus that include infection, immune responses, cell signaling, and apoptosis were modulated by, *E. ictaluri* EseN. Collectively, the above observations support a broader impact of EseN on multiple cellular functions and systems. However, while the above GO analysis provides a general concept for the involvement of broad systems, the analysis does not illustrate details of how these systems are engaged. Therefore, KEGG pathway enrichment analysis was performed by using DAVID. The 13 statistically significant pathways (*p* < 0.05) that were identified are listed in Table 2. Of these, important pathways involved in the modulation of host responses were Toll and Nod receptors signaling pathways, peroxisome pathway, and nucleocytoplasmic transport.

**Figure 3:**
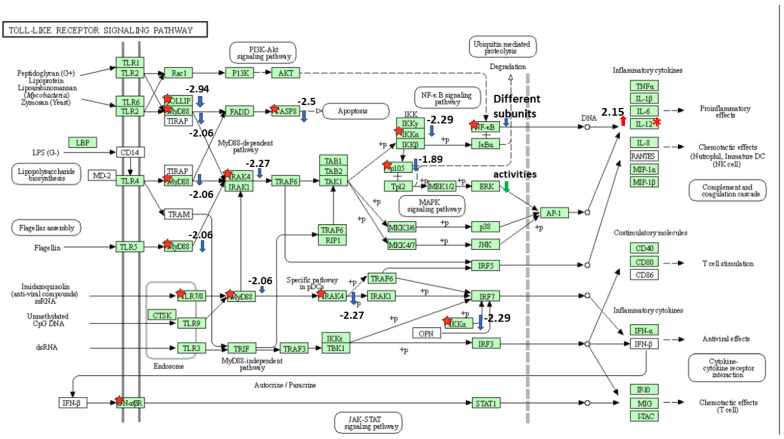
Toll-like receptor signaling pathway. The KEGG pathway enrichment analysis was performed by DAVID. Genes that differently expressed in HKDM-∆EseN mutant compared to HKDM infected by WT indicated by red star and are significant (*p* < 0.05 and q < 0.05). Red arrow indicates up-regulation. Blue arrow indicates downregulation. Numbers indicate log2 fold change in HKDM-∆EseN vs. HKDM-WT.

Because two main pathways that play an important role in the infection were selected as significant, we looked more closely at the genes that were affected by EseN. For the Toll-like receptor signaling pathway (Figure 3), significant DEG were, MYD88 innate immune signal transduction adaptor (*myd88*), caspase 8, component of inhibitor of nuclear factor kappa β kinase complex (*chuk*), cytokine receptor family member b2 (*crfb2*), interleukin-1 receptor-associated kinase 4 (*irak4*), nuclear factor of kappa light polypeptide gene enhancer in B-cells 1 (*nfκβ1*), toll interacting protein (*tollip*), uncharacterized (LOC108266215) and IL12.

For the NOD-like receptor signaling pathway (not illustrated) DEGs were MYD88 innate immune signal transduction adaptor (*myd88*), caspase 8, component of inhibitor of nuclear factor kappa beta kinase complex (*chuk*), cytokine receptor family member b2 (*crfb2*), interleukin-1 receptor-associated kinase 4 (*irak4*), nuclear factor of kappa light polypeptide gene enhancer in B-cells 1 (*nfκβ1*), mitochondrial calcium uniporter (*mcu*), mitofusin2 (*mfn2*), tumor protein p53 binding protein 1 (*tp53bp1*). Both pathways are involved in the regulation of proinflammatory cytokine expression and programmed cell death.

### 3.2. E. ictaluri Effector EseN Modulates Transcription Factor Expression in HKDM

**Figure 4:**
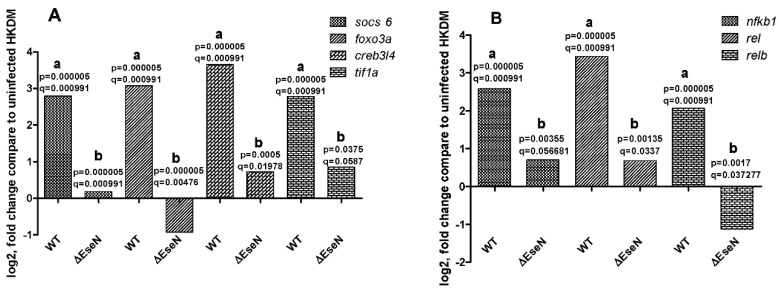
Transcription factor expression. RNAseq data from three independent replicates. (**A**) Not NFκ-β transcription factors. (**B**) Subunits of NFκ-β. Each column represents the mean fold change compared to uninfected HKDM from three independent biological replicates. (WT) HKDM infected with WT, (∆EseN) HKDM infected with ∆EseN. The *p* and q values indicate significance between a: HKDM infected with WT against not infected HKDM, b: HKDM infected with WT, and HKDM infected with ∆EseN mutant.

The RNAseq analysis indicates that many transcription factors were upregulated in HKDM-WT compared to uninfected HKDMs -). But only a few transcription factors were significantly affected by EseN. They include *socs6*, *foxo3a*, *creb3l4*, and *tif1a*. As indicated in Figure 4, all of them were downregulated in HKDM-∆EseN compared to HKDM-WT. This data also indicates that several subunits of NF-κβ were affected by EseN (Figure 4B). Two NF-κβ subunits (*rel1* and *relb*) were significantly downregulated in HKDM-∆EseN compared to HKDM-WT. This data was confirmed by RT-qPCR. 

### 3.3. Effect of E. ictaluri T3SS Effector EseN on Modulation of Pro and Anti-Inflammatory Cytokine Expression in HKDM

The RNAseq data indicate significant (*p* < 0.05 and q < 0.05) upregulation of IL16, IL-12b, IL-8, IL-1b, IL-6, IL-10, IL-15, IL-1a, c-c motif chemokine-3-like, c-c motif chemokine 20 like, c-c motif chemokine 4 homolog, IL-17C, IL-16, CXCL2 like and several others in HKDM-WT. In HKDM-∆EseN mutant compared to HKDM-WT, only IL-16 (*p* = 5.00 × 10^−5^ q = 0.00476143) and IL-12a (*p* = 0.0006249, q = 0.0219) were significantly downregulated and upregulated corresponding. At the same time, significant differences only by *p* value were identified for IL-8 (*p* = 0.00785, q = 0.08968), IL-6 (*p* = 0.0307, q = 0.588), IL-15 (*p* = 0.0405, q = 0.2165) expressions in HKDM-WT compared to HKDM-∆EseN. Therefore, we measured the secretion of pro-inflammatory (IL-8, IL-12a, IL-15, IL-6) cytokines by qPCR at different times after HKDM infection with WT and ∆EseN mutant, and uninfected HKDM as a negative control.

**Figure 5:**
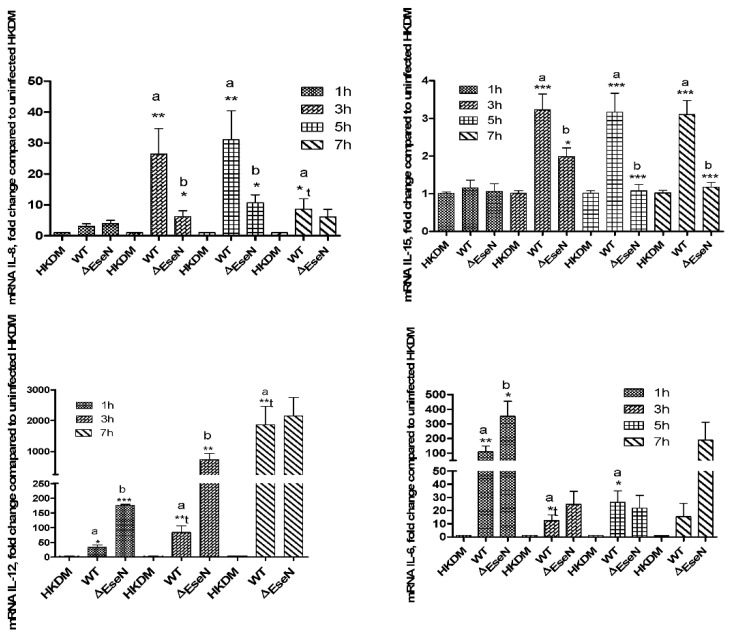
Cytokines expression detected by RT-qPCR. Data were analyzed by Rosh LightCycler^®^ 96 qPCR software using relative expression method. CanX and SDHA were used as reference genes. Comparison between groups was based on One-way ANOVA with the Bonferroni procedure for comparison of group means. Each column represents the mean ± SE of 3 to 4 independent experiments. Comparison between two groups was also analyzed by *t*-test (t); a: indicates significant difference between HKDM-WT (WT) and not infected HKDM (HKDM), b: indicates significant difference between HKDM-WT (WT) and HKDM-∆EseN (∆EseN). * *p* < 0.05, ** *p* < 0.01, *** *p* < 0001.

*E. ictaluri* WT induced IL-8 production in infected HKDM during 3 h, 5 h, and 7 h post-infection (Figure 5). However, cells infected with the ∆EseN mutant strain showed significantly less IL-8 production compared with the WT infected cells at 3 h and 5 h of post-infection testing. There were no differences in production of IL-8 in HKDM-WT compared to those infected with EseN mutant at 1 h and 7 h of post-infection.

During 3 h, 5 h, and 7 h of infection in HKDM-WT production of IL-15 significantly increased compared to uninfected HKDM, and significantly decreased in HKDM-∆EseN compared to HKDM-WT.

The RT-qPCR data indicate that IL-12a is expressed at a low level. Therefore, there were some difficulties to analyze IL-12a expression by RT-qPCR. During 5 h of infection, we were not able to detect expression of IL-12a from uninfected HKDM, IL-12a expression was detected only from HKDM-WT and ∆EesN while expression from HKDM-∆EseN was higher than from cells infected with WT. Our data indicate significant upregulation of IL-12a in HKDM-WT compared to uninfected HKDM at all time points. Significant upregulation of IL-12a was also detected in HKDM-∆EseN mutant compared to HKDM-WT during 1 h and 3 h (Figure 5), of post-infection. This data confirms RNAseq data that demonstrated significant upregulation of IL-12a in HKDM-∆EseN compared to HKDM-WT (log = 2.15, *p* = 0.0006249, q = 0.0219) during 5 h of post-infection.

The HKDM infection with WT bacteria-induced IL-6 production during all time points but at 7 h this increment was not significant (Figure 5). In that same time period, cells infected with the ∆EseN mutant showed significantly higher IL-6 production compared with HKDM-WT only at 1 h of post-infection. At 7 h of post-infection, this difference was not significant (Figure 5).

### 3.4. EseN Modulates COX-2 and Prostaglandin Expression in HKDM

**Figure 6:**
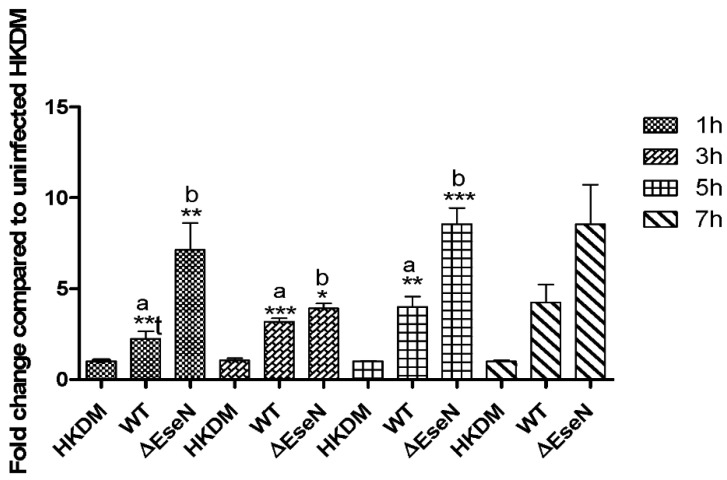
Expression of COX-2 mRNA. Data were analyzed by Rosh LightCycler^®^ 96 qPCR software using relative expression method. CanX and SDHA were used as reference genes. Comparison between groups was based on One-way ANOVA with the Bonferroni procedure for comparison of group means. Comparison between two groups was also analyzed by *t*-test (t); Each column represents the mean ± SE of 3 to 4 independent experiments (depend on time points of infection); a: indicates significant difference between HKDM-WT (WT) and uninfected HKDM (HKDM), b: indicates significant difference between HKDM-WT (WT) and HKDM-∆EseN (∆EseN) * *p* < 0.05, ** *p* < 0.01, *** *p* < 0001.

The inducible form of cyclooxygenase (COX) is COX-2, which forms prostaglandins from arachidonic acid. In response to inflammatory and other physiologic stimuli and growth factors expression of COX-2 is upregulated, and is involved in the production of those prostaglandins that mediate pain and support the inflammatory process. Therefore, we first studied COX-2 mRNA expression in HKDM-WT compared to HKDM-EseN mutant. We used uninfected HKDM as a negative control. The RNAseq data showed that COX-2 mRNA was significantly increased in HKDM-WT compared to uninfected HKDM (log = 2.248, *p* = 0.012, q = 0.012). In HKDM-∆EseN, expression of COX-2 was significantly higher only by *p* value compared to HKDM-WT (log = 1.6, *p* = 0.0255, q = 0.17). Because there was no significance by q value, and to determine COX-2 expression at different time points after infection we performed RT-qPCR at 1 h, 3 h, 5 h, and 7 h post-infection. As illustrated in Figure 6, COX-2 mRNA expression was upregulated in HKDM-WT compared to uninfected HKDM and in HKDM-∆EseN mutant compare to HKDM-WT during all times of post-infection, but significantly only at 1 h, 3 h, and 5 h of post-infection.

Furthermore, we analyzed the ability of COX-2 activity to produce PGE2. The ELISA detection revealed that the PGE2 levels in the culture supernatant were significantly increased in the HKDM-WT and ∆EseN mutant during 3 h, 5 h, and 7 h of post-infection compared to uninfected HKDM. Levels of PGE2 were higher in whole-cell lysates compared to supernatants, indicating that the majority of PGE2 is located inside the HKDM cells. Similarly, for COX-2 mRNA expression, significantly higher synthesis of PGE2 was observed in the HKDM lysates from HKDM infected with ∆EseN mutant compared to cells infected with WT at 1 h, 3 h, and 5 h post-infection.

## 4. Discussions

Using bacterially encoded virulence factors, including an acid-activated urease [32] and a T3SS [33,34] *E. ictaluri* can survive and replicate in channel catfish HKDM [1,32,35]. Subsequent development of infection is dependent on the action of bacterial effector proteins that are translocated into the host cell cytoplasm by the T3SS [2]. Translocated T3SS effector proteins are known to interact with specific host cell target proteins and subvert host defense mechanisms to the pathogens’ benefit by manipulating host cell processes [8,36,37,38,39]. While EseN is a PTL [12], it is probable that its substrate specificity and/or physiological effects differ from its homologs in Shigella (OspF) and Salmonella (SpvC). The proteins share only 63 and 71% AA identity with EseN, which could allow differential specificity towards MAPKs, as well as interaction with different substrates. Differences in timing of translocation and cellular localization following transfection further suggest differential activity. Translocation of OspF occurs from the bacterial cell, through the cytoplasmic membrane, with final location in both the cytoplasm and the nucleus [40]. The SpvC is also translocated through the cytoplasmic membrane by the SPI1 T3SS, but localizes only to the cytoplasm [13]. As reported previously, EseN is translocated from the Edwardsiella containing vacuole (ECV) to the host-cell cytoplasm by the SPI-2-like T3SS of *E. ictaluri* [2,35,41]. These differences suggest activation of different pattern recognition receptors, possible dephosphorylation of different MAPKs, and differential activity in the host cell based on the cellular localization.

Comparative transcriptomic analysis showed a total of 753 DEGs with *p* and q-values of 0.05 or less (Figure 1). A total of 492 from 753 genes were upregulated in HKDM-WT strain and downregulated in HKDM-∆EseN mutant compared to HKDM-WT, indicating a direct role of EseN in the regulation of this gene expression. A total of 254 genes were downregulated only in HKDM-∆EseN, indicating that other *E. ictaluri* effectors or proteins can complement this effect. Transcriptome analyses also indicated differential regulation of several important pathways and their related transcripts in response to *E. ictaluri* effector EseN. Targeting of the Toll-like and, Nod-like receptor pathways and peroxisome pathway by EseN indicates an important role for EseN in the modulation of M1 activation of macrophages [42,43], which leads to activation of protective mechanisms against bacterial infection [44]. Targeting several virus infection pathways that were identified (Table 2), also supports this hypothesis. In addition, M1 macrophages are characterized by the production of pro-inflammatory cytokines, and chemokines, involved in various inflammatory processes [45]. Our data also indicate that EseN affects pro-inflammatory interleukin production (Figure 5). Collectively, our data suggest that EseN is an important player in the modulation of M1 macrophage activation.

The ribosome biogenesis pathway is also targeted by EseN. Bianco and Mohr demonstrated that ribosome biogenesis restricts immune responses to virus infection and DNA [46]. Inhibition of translation is also a well-known strategy that bacteria use to evade immune defense [47].

Several reports indicate that *Salmonella*, *Coxiella*, and *Orientia* counteract innate immune defenses and NF-κβ activation by targeting exportins, importins, or RAN [48,49,50]. Thus, pathogens evolved diverse manners to disturb nucleocytoplasmic traffic to escape innate immunity [48,49,50]. Targeting the nucleocytoplasmic traffic machinery by EseN (Table 2) suggests similar functions.

Targeting Toll-like and NOD-like receptor signaling pathways (Figure 1) leads to modulation of NF-κβ and production of proinflammatory interleukins. The NF-κβ family of transcription factors consists of five genes with similar structures and DNA recognition sequences: p50, p65 (Rel-A), p52, Rel-B, and c-Rel [51]. The nuclear translocation of these transcription factors occurs in response to a wide variety of stimuli leading to the expression of a number of genes related to cell survival and proliferation, cytokine and chemokine production, and coordination of innate and adaptive immune responses to infection [52,53]. Our data indicate that EseN significantly targets the expression of two Rel genes (*c-rel* and *relB*). Inactivation of MAPKs by EseN can lead to up or downregulation of other transcription factors. Our data also indicates that EseN targets the expression of *socs6*, *foxo3a*, *creb3l4*, and *tif1a* transcription factors. Expression mRNA does not indicate transcription factor activity that can depend on post-translational modification and polymerization. Unfortunately, all transcription factors mentioned in this work share 40–60% identity with the same human proteins, so it was impossible to detect their activation.

The key transcription factor of M1 macrophages, NF-κβ, is required for the induction of a large number of inflammatory genes, including interleukins and COX-2 [42]. In this study, we found that *E. ictaluri* EseN modulates the host immune response by increasing IL-8 and IL-15, and reducing IL-12a and IL-6 cytokines during HKDM infection. Interestingly, EseN upregulates IL-8 production during 3 h, 5 h, and 7 h post-infection. This data is opposite to reported data for EseN homologs from *Salmonella* (SpvC) and *Shigella* (OpsF). It was demonstrated that OspF localizes to the host nucleus, and plays a role in remodeling host chromatin, which leads to a reduction of IL-8 transcript. OspF acts for the dephosphorylation/diacylation of histone H3, however, it is unable to dephosphorylate histone H3 in vitro, suggesting that other bacterial or host proteins are required to mediate this process [40]. A *Salmonella* strain expressing SpvC from plasmids shows significantly reduced IL-8 production from infected host cells [13]. It was also demonstrated that IL-8 chemokine production induced by *Salmonella* infection is inhibited by the MEK inhibitor U0126 [54], suggesting that activation of ERK1/2 is required for *Salmonella*-induced inflammation. These differences between *E. ictaluri* EseN and its homologs SpvC and OpsF are unclear. Possibly other T3SS effectors that can interact with different components of the same signaling network to subvert the host response to the infection are involved in this effect [6,8,55,56].

**Figure 7:**
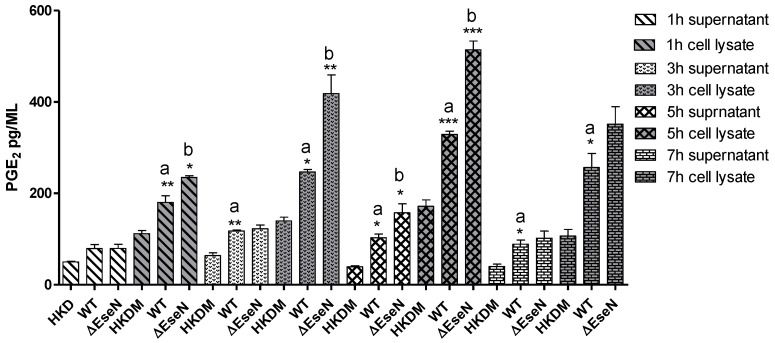
Expression of PGE2. Representative experiment from three independent experiments. Data represents levels of PGE2 expression in uninfected HKDM (HKDM), HKDM-WT (WT), and HKDM-∆EseN mutant (∆EseN). Comparison between groups was based on One-way ANOVA with the Bonferroni procedure. Each column represents the mean ± SE; a: indicates significant difference between HKDM-WT (WT) and uninfected HKDM (HKDM), b: indicates significant difference between HKDM-WT (WT) and HKDM-∆EseN (∆EseN) * *p* < 0.05, ** *p* < 0.01, *** *p* < 0001.

We also demonstrate that WT *E. ictaluri* causes the induction of COX-2 expression in infected HKDM resulting in the upregulation of PGE2 production and that *E. ictaluri* effector EseN inhibits both of these processes (Figure 6 and Figure 7). It has been demonstrated that PGE2 suppresses macrophage production of pro-inflammatory cytokines [57,58] and enhances the synthesis of anti-inflammatory cytokines [59]. These observations lead to the conclusion that PGE2 may participate in the inhibition of the host defense by deactivating macrophage responses against *E. ictaluri*. It was also demonstrated that IL-6 synthesis was stimulated by activation of COX-2 and production of endogenous PGE2 [60]. Our data is consistent with this observation, upregulation of COX-2, IL-6 expression, as well as PGE_2_ production in HKDM-∆EseN mutant, indicates a direct role of EseN in the reduction of COX-2 activity, and IL-6 and PGE2 production. Our data is also consistent with the finding that In *Salmonella* inhibition of ERK1/2 blocks COX-2 expression [57]. It was also reported that *Salmonella* SPI-2 effector *spiC* plays an important role in ERK1/2 activation and therefore activation of Cox-2 expression [57,61]. Interestingly, the inactivation of ERK1/2 by EseN [12] also leads to inhibition of Cox-2 and PEG_2_ expression (Figure 6 and Figure 7). Taken together, these results indicate that EseN causes an ERK1/2 inactivation that leads to decreased COX-2 expression, resulting in the downregulation of PGE2 and IL-6 production in macrophages. This effect is unique to EseN and was not described in its homolog SpvC and Ospf from Salmonella and Shigella, respectively.

Transcription factor FOXO3a is a central transcription factor that mediates multiple physiological and pathological processes by inducing transcription of target genes involved in apoptosis [62], proliferation [63], cell cycle progression [64], survival [65], and DNA damage [66].

In conclusion, the comparative transcriptomic analysis identified 753 genes significantly targeted by *E. ictaluri* T3SS effector EseN that led to modulation of pathways involved in the immune response to infection as identified by Kegg pathway enrichment analysis. The key transcription factor, NF-κβ, is required for the induction of a large number of inflammatory genes, including interleukins and COX-2. Upregulation of NF-κβ would result in the modulation of those inflammatory genes. Indeed, WT *E. ictaluri* induces transcriptional upregulation of IL-8 and IL-15 compared to a ΔEseN mutant, which would result in an enhanced inflammatory response, the recruitment of phagocytes, and phagocytic activation at the site of infection. The inhibition of IL-6 and IL-12a that occurs would result in the inhibition of protective immunity by preventing T- and B-cell growth and development. In addition, the inhibition of PGE2 production by reducing COX-2 transcription could further interfere with the activation, maturation, migration, and cytokine secretion of several immune cells, including macrophages, neutrophils, natural killer cells, and dendritic cells. Overall, the modulating effect of EseN on gene expression in infected HKDM indicates that EseN is essential for successful systemic infection in the catfish host. Importantly, EseN is required for efficient replication in the catfish head kidney and for maximum virulence in the catfish host [12].

## Figures and Tables

**Table 1:** Primers used in this work.

Primers	Forward (5′-3′)	Reverse (5′-3′)
CanX	GCTGTTAAACCGGAGGACTG	GCAGGTCCTCGAAGTAGTCAG
SDHA	CTCCAGGGATGTGGTTTCAC	GCATACAACCCTGGCACTAC
COX2	CAGGTCGAGATGCACTACC	GTAGTAGCCGCTCAGGTG
IL8	GATTGCTCAACCTTTTCGCATTAC	CATGGCCTGTGATTTAGCTGTG
IL6	ACAGCGGAGACACGGA	GGTAGATCCGCTGCAGAC
IL15	CGGCGATTTGTTCGATGCAG	CTCCTGGTTCAAGGGTCAC
IL12a	GAAGACCGTAAGGACCTGTG	TTGCAAAGTAGTAGTGCGGAG
IL16	CATCAGTCGACACCCTGAC	CTGTGGCTTCGCTCCATAC
SOCS6	CGTTGACCTCATCGAGCACT	TCAATCGAACCGGGTACGTG
CREB3L4	GTGGAGTTCTCCGATGCTCA	TCAAGATCAGAGTGGGCGTG
FOXO3a set 1	TGGAGGGACAAGTTGTGTCG	TTGATTAGTGCAGCCAACGGA
FOXO3a set 2	GGAGGGACAAGTTGTGTCGT	AGCCAACGGAAGGCTATTCA
NFKβ1	CGACCCCACTTAACATGGCT	TTGTGGTGGGATGATGACCG
Rel	ACCCTGGGGCTAGTAATGGA	GGGAGGCTGTTTCCACTCTC
RelB	AGACGAGGTCATGAGCACAC	GAGCCACTGTCCTCGTAGTG
TRIM24	GTGCATGGAAGTCGAGGTCT	TTCTCTACGGTGCTGCTTGG

**Table 2:** Pathways affected by *E. ictaluri* effector T3SS EseN.

Category	Term	Gene Count	%	*p* Value
KEGG_PATHWAY	Ribosome biogenesis in eukaryotes	11	1.644245	8.21 × 10^−5^
KEGG_PATHWAY	Nucleocytoplasmic transport	13	1.943199	1.05 × 10^−4^
KEGG_PATHWAY	Herpes simplex virus 1 infection	19	2.84006	0.001088
KEGG_PATHWAY	Lysine degradation	10	1.494768	0.003896
KEGG_PATHWAY	Cell cycle	13	1.943199	0.00414
KEGG_PATHWAY	Measles	12	1.793722	0.009788
KEGG_PATHWAY	Epstein-Barr virus infection	15	2.242152	0.01266
KEGG_PATHWAY	One carbon pool by folate	4	0.597907	0.020781
KEGG_PATHWAY	Toll and Imd signaling pathway	6	0.896861	0.02225
KEGG_PATHWAY	Toll-like receptor signaling pathway	9	1.345291	0.022977
KEGG_PATHWAY	Peroxisome	8	1.195815	0.026114
KEGG_PATHWAY	NOD-like receptor signaling pathway	11	1.644245	0.029469
KEGG_PATHWAY	Alcoholic liver disease	10	1.494768	0.043176

## Data Availability

Data is provided in this article.
